# Constructing a meteorological indicator dataset for selected European NUTS 3 regions

**DOI:** 10.1016/j.dib.2020.105786

**Published:** 2020-05-29

**Authors:** Denitsa Angelova, Norman Blanco Lupio

**Affiliations:** a Technical University of Munich, School of Management; b Technical University of Munich, School of Life Sciences Weihenstephan

**Keywords:** Meteorological data, Weather data, Weather extremes, Extreme weather, Weather volatility, Climate change, Heat wave, Drought

## Abstract

The harmonization of data granularity in spatial and temporal terms is an important pre-step to any econometric and machine learning applications. Researchers, who wish to statistically test hypotheses on the relationship between agro-meteorological and European policy outcomes, often observe that agro-meteorological data is typically stored in gridded and temporally detailed form, while many relevant policy outcomes are only available on an aggregated level. This dataset intends to aid empirical investigations by providing a dataset with monthly meteorological indicators on a European Nomenclature of Territorial Units for Statistics level 3 (NUTS 3) regional level for 13 countries for the period from 1989 to 2018. The data we provide allows researchers to investigate hypothesis related to weather volatility and the probability of extreme weather events.

We created this dataset from the daily data in grids of 25 km x 25 km provided by the Joint Research Centre of the European Commission. We matched the map with the raw data to a map with the administrative boundaries of European NUTS 3 regions. After appropriately weighting, we calculated the monthly, regional mean, variance and kurtosis of the following variables: maximum, minimum, average air temperature in degrees Centigrade, sum of precipitation in mm and snow depth in cm. We report the covariance between the average temperature and the precipitation as well.

Specifications table

**Table:** 

Subject	Economics and econometrics
Specific subject area	Policy evaluation related to soil protection, environment, agriculture, food security, energy, climate change, health and sustainable development
Type of data	Table (26 Tables)
How data were acquired	The raw gridded agro-meteorological data was obtained through the public data repository of the Joint Research Centre of the European Commission. The analysis of the gridded agro-meteorological data was performed in the GIS software ArcMap version 10.5.
Data format	Raw Analyzed
Parameters for data collection	Weighting parameter: a NUTS 3 region would typically be fragmented in multiple grid cells. In order to arrive at a value at the NUTS 3 level we calculate a weighted sum of the values of individual grid cells. The weights are proportionate to the area covered by the cells within a specific NUTS 3 region.
Description of data collection	We calculated the NUTS 3 (Nomenclature of Territorial Units for Statistics level 3) dataset from the gridded agro-meteorological data provided by the Joint Research Centre (JRC) of the European Commission (EC) [1]. The gridded data has been georeferenced to the NUTS 3 boundaries using GIS software. The calculated intersections between the two maps are used to calculate the monthly mean, variance and kurtosis of the variables in the gridded dataset on a NUTS 3 level. The covariance between average temperature and precipitation is also calculated.
Data source location	Country: Austria, Belgium, Denmark, France, Germany, Italy, Luxembourg, Netherlands, Poland, Portugal, Spain, Sweden, United Kingdom
Data accessibility	Both raw and analyzed data are available on a public repository. Raw data Repository name: Mendeley Data Data identification number: 10.17632/csbx34pzsy.1 Direct URL to data: http://dx.doi.org/10.17632/csbx34pzsy.1 Analyzed data Repository name: Mendeley Data Data identification number: 10.17632/sf9x4h5jfk.3 Direct URL to data: http://dx.doi.org/10.17632/sf9x4h5jfk.3

## Value of the data

• Policy and economic outcomes are often observed on regional level, while meteorological data is typically provided in gridded, spatially disaggregated form. This dataset, in contrast, provides meteorological indicators on an administrative regional level. It is useful for the investigation of climate and weather effects on phenomena observed at this regional level.
• Main beneficiaries of the data include applied researchers working on issues related to soil protection, environment, agriculture, food security, energy, climate change, health and sustainable development.
• The dataset is useful for machine learning and econometric applications related to weather. It provides potential features for predictive machine learning applications, potential regressors in econometric applications testing the significance of weather effects or quantifying the interaction effects of weather and other explanatory variables on a dependent variable.

## 1 Data Description

The repository hosts 26 comma-separated tables, 13 tables with raw data and 13 tables with analysed data. Each of the tables corresponds to one of the 13 countries under investigation: Austria (AT), Belgium (BE), Denmark (DK), France (FR), Germany (DE), Italy (IT), Luxembourg (LU), Netherlands (NL), Poland (PL), Portugal (PT), Spain (ES), Sweden (SE), United Kingdom (UK). The country the data refers to can be inferred from the title of the data file, while the structure of the tables is identical for each country.

The raw data consists of daily observations for 25 km x 25 km grid cells on the following variables:

– daily maximum air temperature in degrees Centigrade,
– daily minimum air temperature in degrees Centigrade,
– daily average air temperature in degrees Centigrade,
– sum of precipitation in mm per day,
– snow depth in cm.

The analysed data reports monthly, regional moments (mean, variance and kurtosis) of the distributions. The covariance between the daily average temperature and the precipitation are reported as well.

Each table with analysed data contains the following columns (header in brackets):

– Observation Index (“”): an number identifying the observation,
– NUTS 3 code (“NUTS”): NUTS 3 code, e.g. “DE212” for the German district of Munich,
– Year (“YEAR”): year the observation refers to,
– Month (“MONTH”): month the observation refers to,
– Mean of max temperature ("Mean_MAX_T"): monthly mean of maximum air temperature,
– Mean of min temperature ("Mean_MIN_T"): monthly mean of minimum air temperature,
– Mean of average temperature ("Mean_AVG"): monthly mean of average air temperature,
– Mean of precipitation ("Mean_Pre"): monthly mean of sum of precipitation,
– Mean of snow depth ("Mean_Snow"): monthly mean of snow depth,
– Variance of max temperature ("Var_MAX_T"): monthly variance of maximum air temperature,
– Variance of min temperature ("Var_MIN_T"): monthly variance of minimum air temperature,
– Variance of average temperature ("Var_AVG"): monthly variance of average air temperature,
– Variance of precipitation ("Var_Pre"): monthly variance of daily sum of precipitation,
– Variance of snow depth ("Var_Snow"): monthly variance of snow depth,
– Kurtosis of max temperature ("Kur_Max"): monthly kurtosis of daily maximum air temperature,
– Kurtosis of min temperature ("Kur_Min"): monthly kurtosis of daily minimum air temperature,
– Kurtosis of average temperature ("kur_mean"): monthly kurtosis of daily average air temperature,
– Kurtosis of precipitation ("kur_pre"): monthly kurtosis of daily sum of precipitation,
– Kurtosis of snow depth ("kur_Snow"): monthly kurtosis of snow depth,
– Covariance between average temperature and precipitation ("Cov_Temp_Pre"): monthly covariance between average air temperature and precipitation sum.

## 2 Experimental Design, Materials and Methods

We downloaded the gridded agro-meteorological data [1] for each of the thirteen countries. We than georeferenced the values to the NUTS 3 and calculated the intersections using the GIS software ArcMap 10.5 with the help of two maps (shapefiles): one containing a grid of 25 km x 25 km and one containing the NUTS 3 boundaries. It is important to verify that the maps have the same projection before computing the intersection, in this case the Lambert Azimuthal Equal Area projection. A NUTS 3 region is typically fragmented in multiple grid cells. To arrive at a value at the NUTS 3 level we calculate a weighted sum of the values of individual grid cells. The weights are proportionate to the area covered by the cells within a specific NUTS 3 region. We use the interpolated daily NUTS 3 values to calculate the monthly moments of the distributions of the random variables as well as the covariance between the daily average temperature and the daily precipitation.

## Declaration of Competing Interest

The authors declare that they have no known competing financial interests or personal relationships which have, or could be perceived to have, influenced the work reported in this article.

## Appendix Supplementary materials

Image, application 1
